# Comparative effects of glazing versus polishing on mechanical, optical, and surface properties of zirconia ceramics with different translucencies

**DOI:** 10.1002/cre2.884

**Published:** 2024-05-26

**Authors:** Maryam Jamali, Fariba Ezoji, Behnaz Esmaeili, Soraya Khafri

**Affiliations:** ^1^ Student Research Committee Babol University of Medical Sciences Babol Iran; ^2^ Department of Restorative Dentistry, Dental Materials Research Center, Health Research Institute Babol University of Medical Sciences Babol Iran; ^3^ Department of Biostatistics and Epidemiology, School of Public Health Babol University of Medical Sciences Babol Iran

**Keywords:** flexural strength, surface properties, zirconium oxide

## Abstract

**Objectives:**

This study compared the effects of glazing versus polishing on mechanical, optical, and surface properties of zirconia ceramics with different translucencies.

**Materials and Methods:**

In this in vitro study, 120 bar‐shaped specimens (25 × 4 × 1.2 mm) were fabricated from three different types of zirconia with different translucencies (*n* = 40, DD Bio ZW, ZX2, and Cube X2). After sintering, each zirconia group was randomly divided into five subgroups of control (glazing), glazing + bur abrasion, glazing + bur abrasion + polishing with EVE Diacera® kit, glazing + bur abrasion + reglazing, and glazing + bur abrasion + polishing with EVE Diacera® kit + reglazing. The specimens underwent surface roughness, hardness, flexural strength, and translucency tests, as well as X‐ray diffraction (XRD) and scanning electron microscopy (SEM) for assessment of surface topography. Data were analyzed by one‐way analysis of variance, Tukey test, and Pearson test (*α* = .05).

**Results:**

Flexural strength, surface hardness, and translucency were significantly correlated with zirconia type. ZW zirconia had significantly higher flexural strength and surface hardness and significantly lower translucency than Cube X2 and ZX2 (*p* < .001). Surface roughness had no significant correlation with zirconia type (*p* = .274). Polishing created the smoothest, and bur abrasion created the roughest surface (*p* < .001). Flexural strength and hardness in most experimental groups were significantly lower than in the control group (*p* < .001). Translucency was not significantly different in bur abrasion and polishing groups, compared with the control group; however, reglazing significantly increased the translucency (*p* < .001). SEM micrographs confirmed the surface roughness results. XRD showed monoclinic phase only in reglazed groups.

**Conclusion:**

Of different surface treatments, polishing improved the surface properties and caused the smallest change in mechanical properties of zirconia with different translucencies.

## 1 INTRODUCTION

At present, due to the high demand for esthetic restorations, metal‐free restorations are the topic of several investigations (Gracis et al., 2015; Pereira et al., 2015). Polycrystalline monolithic zirconia has been suggested for the fabrication of high‐strength restorations with optimal esthetics. Zirconia has high wear resistance, flexural strength, and toughness. It also possesses favorable biocompatibility and no metal component to cause allergic reactions, chemical corrosion, or discoloration. Also, wear of the opposing teeth by zirconia is lower than that caused by feldspathic porcelain (Park et al., 2017). Two types of zirconia restorations are currently used: porcelain veneered zirconia (feldspathic porcelain with zirconia coping) and zirconia with anatomical contour (monolithic zirconia) which only contains zirconia (Park et al., 2017).

Zirconia is an opaque material, and the opacity reduces its esthetic qualities therefore different methods are available for the improvement of translucency of yttrium‐stabilized tetragonal zirconia (Y‐TZP) ceramics, such as increasing the yttria content, transformation to cubic zirconia phase, and reduction of aluminum oxide content. These strategies affect the crystalline stability of zirconia and subsequently its mechanical properties. Although the efficacy of different polishing systems for porcelain restorations has been the topic of numerous investigations, information regarding the efficacy of zirconia polishing methods is limited (Gracis et al., 2015; Pereira et al., 2015).

Moreover, the effects of abrasion and polishing on mechanical, surface, and optical properties of high‐translucency zirconia restorations and their long‐term clinical service have yet to be elucidated. Further preservation of tooth structure has led to the fabrication of thinner restorations than the conventional types. In the posterior areas with heavier masticatory forces, polycrystalline ceramics can be the first choice due to their transformation toughening, which does not occur in other all‐ceramic restorations (Park et al., 2017).

Veneering porcelain is added to zirconia coping for the improvement of esthetics (De Souza et al., 2020). Although fracture of zirconia coping rarely occurs, fracture of the feldspathic porcelain veneering occurs more frequently therefore the utilization of monolithic zirconia with high translucency has increased for its esthetic appeal and higher fracture resistance compared to zirconia restorations layered with feldspathic porcelain (Sulaiman et al., 2016). Despite the advances in computer‐aided design/computer‐aided manufacturing technology, intraoral adjustment of restorations is still imperative for optimal occlusion, and also to ensure emergence profile (Guilardi et al., 2017; Park et al., 2017). Due to the high surface hardness of zirconia, diamond burs are used for clinical adjustment, which may eliminate the glaze layer and impair the surface smoothness (Etman et al., 2008). Roughness and irregularity of the restoration surface can lead to severe wear of the opposing enamel or restoration (Caglar et al., 2018). Surface roughness may also lead to plaque accumulation, gingival inflammation, dental caries, and periodontal disease (Hmaidouch et al., 2014; Rashid, 2014).

The intraoral polishing systems were introduced as a replacement for reglazing and are imperative to prevent or minimize fast wear of the opposing teeth. Moreover, polishing increases the durability and improves the esthetic appearance of restorations by the elimination of defects caused by wear (Caglar et al., 2018; Preis et al., 2011). Since there is no standard protocol for the treatment of monolithic zirconia, especially high‐translucency zirconia, after sintering, this study aimed to compare the effects of glazing and/or polishing on mechanical, optical, and surface properties of zirconia ceramics with different translucencies. The null hypothesis of the study was that the type of zirconia and surface treatment would have no significant effect on flexural strength, surface hardness, surface roughness, and translucency of zirconia ceramics.

## 2 MATERIALS AND METHODS

This in vitro experimental study was carried out on 120 (Zucuni et al., 2017) bar‐shaped zirconia specimens with 25 mm length, and 4 mm width, 1.2 mm thickness (Hjerppe et al., 2016) fabricated from different types of zirconia with different translucencies. The study protocol was approved by the ethics committee of Babol University of Medical Sciences (IR.MUBABOL.HRI.REC.1401.157).

### 2.1 Sample size

The sample size of the study was calculated to be eight specimens in each group (a total of 120) according to a previous study (Hjerppe et al., 2016) using G* Power software (version 3.0.10). To discern a 5% disparity with an effect size of 1.72, it was necessary to have a sample size of eight specimens in each group with 80% power.

### 2.2 Specimen preparation

Bar‐shaped zirconia specimens were obtained by sectioning of DD Bio ZW, DD Bio ZX2, and DD Bio Cube X2 (Dental Direct) zirconia blanks by a precision‐sectioning machine (Mashhad, Iran). The specimens initially had 30 mm length, 1.4 mm thickness, and 5 mm width. They were then polished with 800‐grit abrasive paper (Schatz et al., 2016) and sintered. After sintering, the specimens reached their final dimensions of 25 mm length, 1.2 mm thickness, and 4 mm width considering the scaling factor of 1.234 (Yang et al., 2020). The upper surface of sintered specimens was sandblasted with 50 µm alumina powder at 30 Psi pressure, and they were then dried at room temperature. The glaze paste and liquid were mixed and applied on the sandblasted surface, and the specimens were heated in a ceramic furnace to obtain a glazed surface. The specimens were then sintered and glazed as instructed by the manufacturer and were randomly assigned to the following five groups:

*Group* 1: Control group (sintering + glazing); Group 2: sintering + glazing + diamond bur abrasion; Group 3: sintering + glazing + diamond bur abrasion + polishing with EVE Diacera® polishing kit; Group 4: sintering + glazing + diamond bur abrasion + reglazing; Group 5: sintering + glazing + diamond bur abrasion + polishing with EVE Diacera® polishing kit + reglazing.

### 2.3 Sintering

For sintering of specimens as instructed by the manufacturer, the zirconia specimens were initially heated to 900°C and remained at this temperature for 30 min. They were then heated to 1450°C and remained at this temperature for 120 min, and were then cooled down to 200°C (scaling factor: 1.234).

### 2.4 Glazing

After the application of glaze material (UniGlaze; Dental Direkt), the zirconia specimens were first placed in a furnace (Certified VITA furnances) for 2 min with a baseline glazing temperature of 440°C and a heating rate of 45°C/min until the temperature reached 815°C. The specimens remained at this temperature for 1 min. The cooling time was 3 min.

### 2.5 Surface treatments

The control specimens did not undergo any additional surface treatment. The remaining groups were abraded with 46 µm red‐band diamond bur (D+Z) with a high‐speed hand‐piece operating at 400,000 rpm under water coolant for 30 s. Horizontal movements in the same direction were applied for this purpose. A new bur was used for each specimen. For polishing of zirconia specimens, the EVE Diacera® Zirconia Polishing Kit was used with the following order:

1. code:H2DCmf/particle size: medium grain.
2. code:H2DCf/particle size: fine grain.

Polishing was performed with low‐speed hand‐piece for 1 min for each specimen.

### 2.6 Surface hardness

The surface hardness of specimens was measured by a Vickers hardness tester (MH1 model) by applying a 4.9 N load within 20 s by a diamond‐shaped pyramid indenter. The results were reported quantitatively.

### 2.7 Surface roughness

The surface roughness was measured by a profilometer (Fanavaran Pars Nemo Iran, Delta) by measuring the Ra value. Three measurements were made for each specimen, and the mean of the three values was calculated and reported.

### 2.8 The 3‐point bending test

Bar‐shaped specimens were placed on a steel jig with 1.6 mm diameter and 15 mm distance from each other in a universal testing machine (TB‐5T; Koopa). The load was applied by a plunger with a 1.6 mm diameter at a crosshead speed of 1 mm/min until fracture of zirconia.

The 3‐point flexural strength was calculated using the following formula:

$$\sigma =3Nl/(2{\text{bd}}^{2}),$$

where N is the fracture load in Newtons (N), *l* is the distance between the supports in millimeters, *b* is the width of specimen in millimeters, and *d* is the thickness of specimens in millimeters.

### 2.9 Assessment of surface topography

One random specimen from each group was inspected under a scanning electron microscope (SEM; SNE‐4500M, South Korea) after surface treatment. For this purpose, the specimens were first gold‐coated and then underwent SEM.

### 2.10 Assessment of crystalline structure

One random specimen from each group underwent X‐ray diffraction (XRD; D8 Advance; Bruker) to assess the crystalline structure and monoclinic and tetragonal phases after surface treatments.

### 2.11 Assessment of translucency

The translucency of all specimens was measured before the flexural strength test by using a spectrophotometer (Vita Easy Shade; Vita ZahnFabrik) against white‐and‐black backgrounds, by assessing the right and left side of each specimen. Before measurements, the spectrophotometer was calibrated as instructed by the manufacturer. The degree of translucency was measured using the following formula:

$$\text{TP}={\left[{\left(L_{b}-L_{w}\right)}^{2}+{\left(a_{b}-a_{w}\right)}^{2}+{\left(b_{b}-b_{w}\right)}^{2}\right]}^{1/2},$$

where w indicates the white and b indicates the black background.

### 2.12 Statistical analysis

The Shapiro–Wilk test was used to assess the normality of data distribution, which showed normal distribution of data. The homogeneity of the variances was also confirmed by the Levene's test. Thus, data were compared by two‐way analysis of variance (ANOVA) followed by sequential one‐way ANOVA. Subsequently, Tukey's post hoc Honest Significant Difference test was applied for pairwise comparisons. All statistical analyses were performed using SPSS version 25 (IBM Corp.) at the 0.05 level of significance. This information was added to the text.

## 3 RESULTS

### 3.1 Surface roughness

Table 1 presents the mean surface roughness (Ra in nm) of the five surface treatment groups of the three types of zirconia. The results revealed significant differences among subgroup 4 of the three types of zirconia (*p* < .001). However, the difference among subgroups 1, 2, 3, and 5 of the three zirconia types was not significant. The interaction effect of zirconia type and surface treatment type was significant on the mean surface roughness of the five subgroups of the three zirconia types (*p* < .001).

**Table 1:** Mean surface roughness (Ra in nm) of the five surface treatment groups of the three types of zirconia.

Treatment/Zirconia	1	2	3	4	5	*p* Value
DD BIO ZW	66.5 ± 20.5^aA^	124.1 ± 40.5^bA^	21.2 ± 12^aA^	64.9 ± 33^aA^	57.5 ± 26.1^aA^	<.001
DD BIO ZX2	37.9 ± 20.9^acA^	155.7 ± 26.8^bA^	22.9 ± 15.7^cA^	66.1 ± 25.1^aA^	68.3 ± 31.8^aA^	<.001
DD BIO CUBEX2	46.8 ± 17.2^aA^	128.8 ± 52^bA^	21.8 ± 12.6^aA^	92.2 ± 55^cB^	64.3 ± 30.8^aA^	<.001
*p* Value	.259	.274	.968	.001	.268	<.001*

*Note*: Comparisons among the five subgroups are shown with uppercase letters, while comparisons among the three zirconia types are shown with lowercase letters. Similar letters indicate the absence of a significant difference, while dissimilar letters indicate the presence of a significant difference.

The level of significance was set at 0.05.

* Interaction effect of zirconia type and surface treatment type with two‐way analysis of variance.

### 3.2 Surface hardness

Table 2 shows the mean Vickers surface hardness (kgf/mm^2^) of the five surface treatment groups of the three types of zirconia. Comparison of the three types of zirconia showed the absence of a significant difference in surface hardness only in subgroup 2 (*p* > .05), and significant differences were found among the three zirconia types in all other surface treatments (*p* < .001).

**Table 2:** Mean Vickers surface hardness (kgf/mm^2^) of the five surface treatment groups of the three types of zirconia.

Treatment/Zirconia	1	2	3	4	5	*p* Value
DD BIO ZW	1417.4 ± 21.4^aA^	1084.6 ± 36.9^bA^	1398.1 ± 34.5^acA^	1340.9 ± 33.5^cA^	1227 ± 44.9^dA^	<.001
DD BIO ZX2	1375.5 ± 32.9^aAB^	1092.6 ± 54.9^bA^	1269 ± 47.5^cB^	1273.7 ± 33.8^cA^	1091.6 ± 55.1e^bB^	<.001
DD BIO CUBEX2	1338.1 ± 89.6^aB^	1088.2 ± 74.4^bA^	1286.1 ± 70.2^aB^	1256.9 ± 52.4^aB^	1096.9 ± 52.4^bB^	<.001
*p* Value	.038	.962	.001	<.001	.001	<.001*

*Note*: Comparisons among the five subgroups are shown with uppercase letters, while comparisons among the three zirconia types are shown with lowercase letters. Similar letters indicate the absence of a significant difference, while dissimilar letters indicate the presence of a significant difference.

Level of significance was set at 0.05.

* Interaction effect of zirconia type and surface treatment type with two‐way analysis of variance.

### 3.3 Flexural strength

Table 3 presents the mean flexural strength of the five surface treatment groups of the three types of zirconia. No significant difference was found in the flexural strength of the fifth subgroup of the three types of zirconia (*p* > .05). The difference among the three zirconia types in the remaining four subgroups was significant (*p* < .001). The interaction effect of zirconia type and surface treatment on the flexural strength of zirconia was significant (*p* < .001).

**Table 3:** Mean flexural strength (MPa) of the five surface treatment groups of the three types of zirconia.

Treatment/Zirconia	1	2	3	4	5	*p* Value
DD BIO ZW	1318.7 ± 109.1^aA^	1052.5 ± 21.7^bA^	981.2 ± 99.1^bA^	1061.2 ± 91.6^bA^	1010.6 ± 128.3^bA^	<.001
DD BIO ZX2	952.5 ± 46^aB^	826.2 ± 48.7^bB^	847 ± 64.2^abB^	793.7 ± 55.8^bB^	935.6 ± 104.1^aA^	<.001
DD BIO CUBEX2	872.5 ± 77.1^aB^	711.5 ± 87.2^bB^	917.5 ± 126.6^aAB^	980.4 ± 91.1^aA^	974.9 ± 62.2^aA^	<.001
*p* Value	<.001	<.001	.045	<.001	.357	<.001*

*Note*: Comparisons among the five subgroups are shown with uppercase letters, while comparisons among the three zirconia types are shown with lowercase letters. Similar letters indicate the absence of a significant difference, while dissimilar letters indicate the presence of a significant difference.

The level of significance was set at 0.05.

* Interaction effect of zirconia type and surface treatment type with two‐way analysis of variance.

### 3.4 Translucency

Table 4 presents the translucency of the five surface treatment groups of the three types of zirconia. Significant differences existed in translucency of the three zirconia types in all five surface treatments (*p* < .001). The interaction effect of zirconia type and surface treatment on the mean translucency was significant (*p* < .001).

**Table 4:** Translucency of the five surface treatment groups of the three types of zirconia.

Treatment/Zirconia	1	2	3	4	5	*P* value
DD BIO ZW	8.8 ± 0.2^aC^	8.8 ± 0.3^aC^	8.6 ± 0.2^aA^	9.5 ± 0.3^bA^	9.3 ± 0.3^abA^	<.001
DD BIO ZX2	9.5 ± 0.3^aB^	9.1 ± 0.4^aA^	9.3 ± 0.3^aB^	10.6 ± 0.4^bB^	10.4 ± 0.5^bB^	<.001
DD BIO CUBEX2	11.4 ± 0.3^aC^	10.6 ± 0.4^bB^	10.6 ± 0.4^cbC^	12 ± 0.3^dC^	11.5 ± 0.3^aC^	<.001
*p* Value	<.001	<.001	<.001	<.001	<.001	<.001*

*Note*: Comparisons among the five subgroups are shown with uppercase letters, while comparisons among the three zirconia types are shown with lowercase letters. Similar letters indicate the absence of a significant difference, while dissimilar letters indicate the presence of a significant difference.

* Interaction effect of zirconia type and surface treatment type with two‐way analysis of variance.

### 3.5 XRD

**Figure 1:**
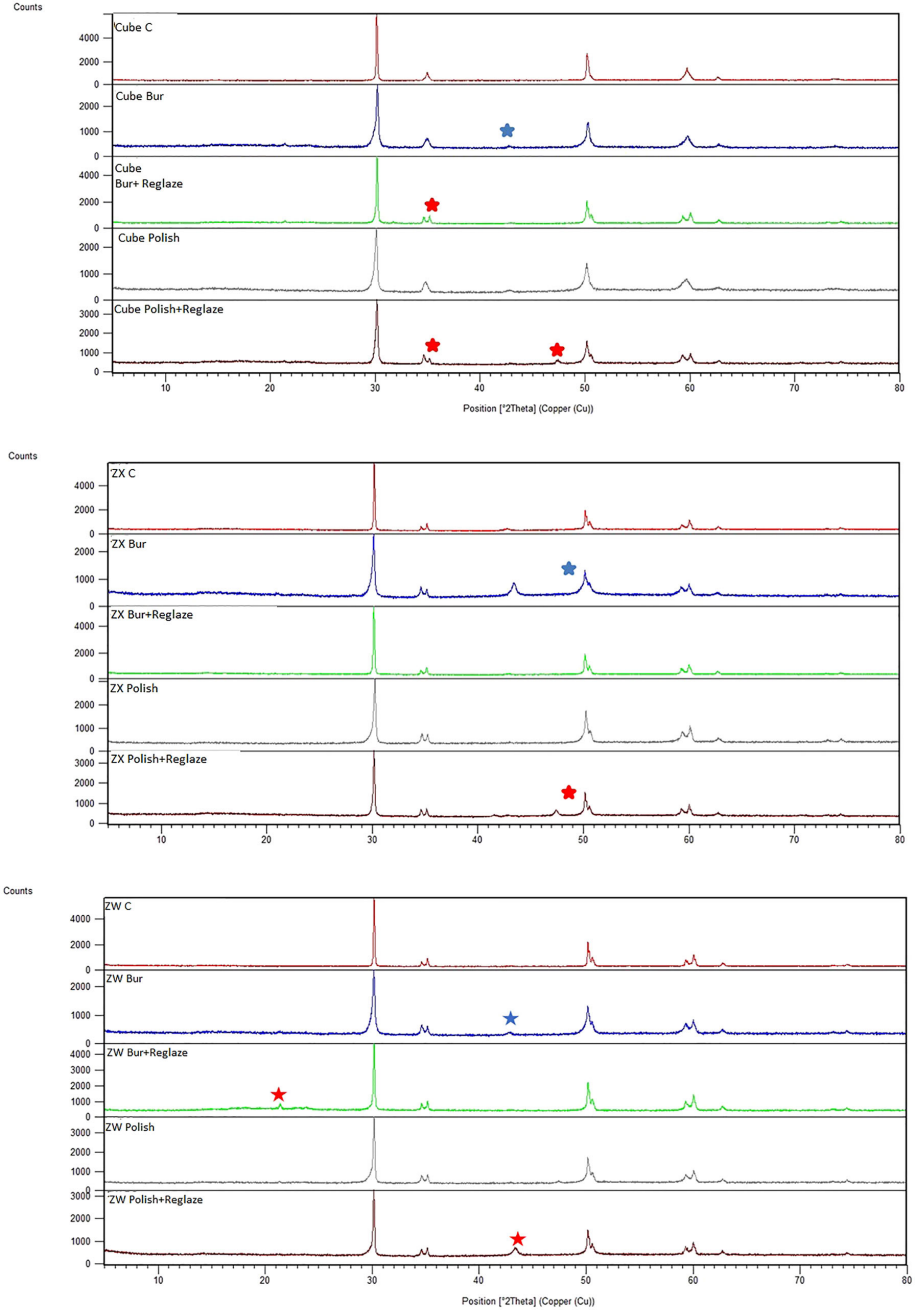
XRD pattern of the three types of zirconia subjected to different surface treatments. Red stars indicate the presence of monoclinic phase in reglazed specimens. Blue star indicates zirconia phase transformation in bur abrasion + reglazing group. The peak related to bur has been eliminated in this group. XRD, X‐ray diffraction.

Figure 1 shows the XRD pattern of the three types of zirconia subjected to different surface treatments. The monoclinic phase was seen in reglazed specimens.

### 3.6 SEM

**Figure 2:**
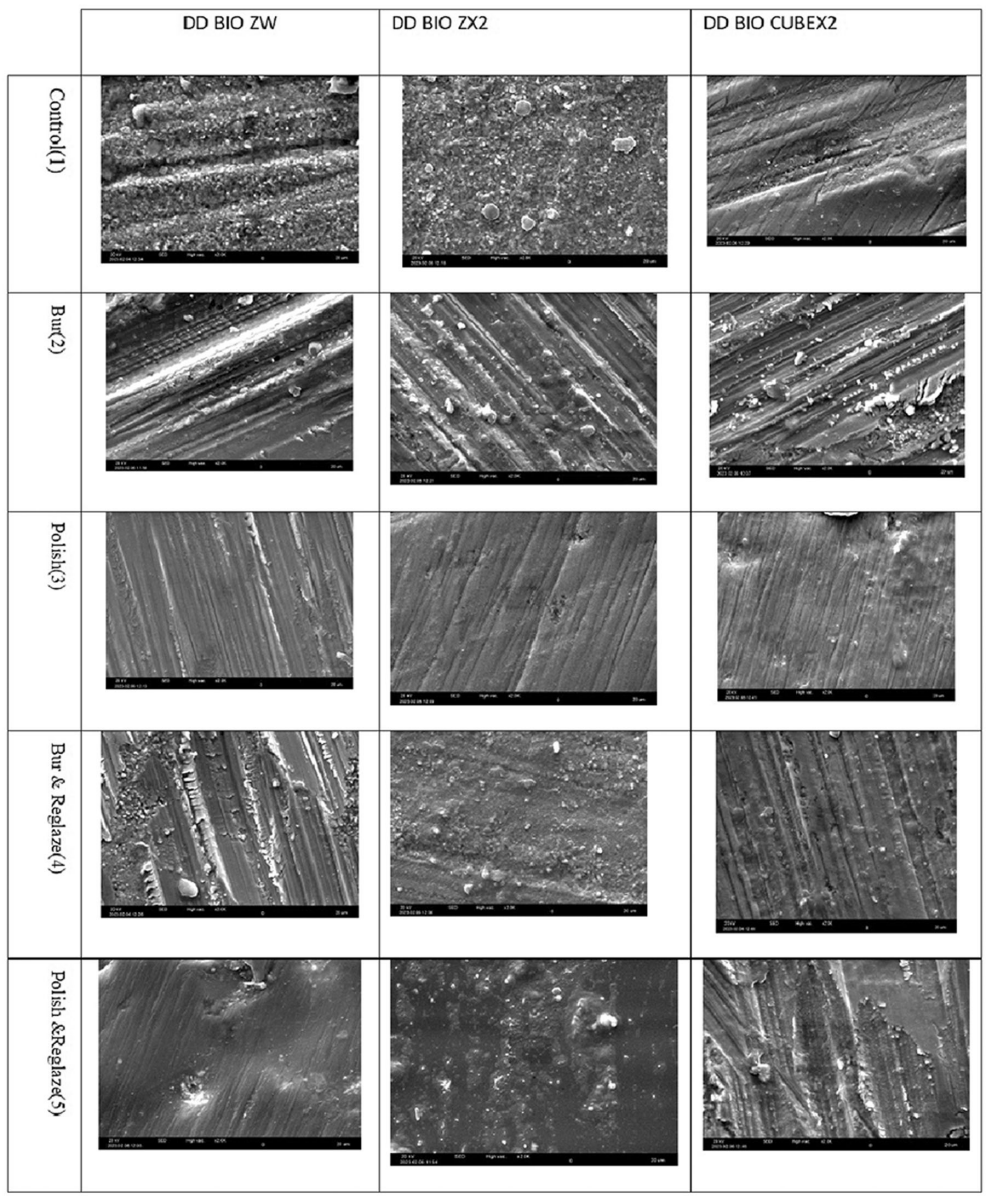
Scanning electron microscopy photomicrographs of the groups.

Figure 2 presents the SEM photomicrographs of the groups.

As seen in the images, the Bur subgroup (2) has the highest surface roughness, and the polished subgroup (3) shows the smoothest surface. Reglazed subgroups (4, 5) also show an increase in roughness.

## 4 DISCUSSION

This study compared the effects of glazing and/or polishing on the mechanical, optical, and surface properties of zirconia ceramics with different translucencies. The null hypothesis of the study was that the type of zirconia and surface treatment would have no significant effect on flexural strength, surface hardness, surface roughness, and translucency of zirconia ceramics. According to the obtained results, the null hypothesis of the study regarding flexural strength, surface hardness, and translucency was rejected but the part of null hypothesis regarding the effect of zirconia type on surface roughness was almost accepted.

Regarding flexural strength, the results showed that the zirconia type with lower translucency (ZW) in most surface treatment subgroups had significantly higher flexural strength than the other two types of zirconia with higher translucency (ZX and Cube X2). The surface hardness of ZW zirconia with lower translucency in most surface treatment subgroups was significantly higher than that of ZX and Cube X2 zirconia types with higher translucency. Translucency had a significant correlation with most surface treatments. The surface roughness had no significant correlation with the type of surface treatment in any zirconia type.

The present results showed that zirconia with lower translucency (ZW) in most surface treatment subgroups had significantly higher flexural strength than the other two types of zirconia with higher translucency (ZX and Cube X2). Cubic zirconia with higher yttria content has lower transformation toughening, resulting in a significantly lower flexural strength (Zhang et al., 2016). The flexural strength results of the present study were in agreement with those of Singh et al. (2021) and Kumchai et al. (2018). They showed that glazing of high‐translucent zirconia significantly decreased the flexural strength at the corner of several specimens. These sites could serve as the sites of initiation of fracture due to the formation of voids in the glaze layer at the glaze/zirconia interface. The voids in the glaze layer could have been resulted from a mismatch between the coefficients of thermal expansion of monolithic zirconia and glaze layer in the process of cooling. Ozturk et al. (2022) and some others (Fairhurst et al., 1992; Yener et al., 2011) showed that glazing decreased the flexural strength, which may be due to the formation of monoclinic phase with poor mechanical properties at high glazing temperatures, and also reverse transformation. The same result was obtained in glazed subgroups of the present study (Singh et al., 2021). Some researchers (Denry & Kelly, 2008; Sundh et al., 2005) showed that heating of Y‐TZP at 900°C for 1 h or 900–1000°C for 1 min would cause reverse transformation and reduction in flexural strength, due to reverse transformation. Another reason for poor mechanical properties is that glazing causes porcelain cracking and flexural strength reduction (Fairhurst et al., 1992). In total, changes in microstructure and composition of zirconia for enhancement of its translucency can affect its mechanical resistance and flexural strength (Dal Piva et al., 2018).

The present results showed that the surface hardness of ZW zirconia with lower translucency was significantly higher than the other two types of zirconia with higher translucency in most surface treatments. Stawarczyk et al. (2013), Pereira et al. (2015), Nam and Park (2017), and Sen et al. (2018) found no significant difference in surface hardness of different zirconia types. Klimke et al. (2011) and Sen et al. (2018) reported controversy in the surface hardness of zirconia types and attributed the differences to the type of material, phase content, and sintering temperature. On the other hand, zirconia with higher yttria percentage is more susceptible to bulk fracture probably due to lower surface hardness of cubic zirconia and ZX, compared with ZW (Jum'ah et al., 2020). Grinding with diamond burs significantly changes the surface hardness. Polishing decreases the surface hardness, as expected (Karakoca & Yılmaz, 2009; Mohammadi‐Bassir et al., 2017).

In the present study, bur abrasion significantly increased the surface roughness compared with the control group; however, this increase was not significantly different among the three ceramic types. Jum'ah et al. (2020) evaluated the effect of polishing on zirconia layers with different translucencies. Consistent with the present study, their results showed that grinding or bur abrasion significantly increased the surface roughness of all specimens.

Clinical adjustment was standardized in the present study, including the frequency of abrasive movements with bur for each specimen, speed of hand‐piece, duration of adjustment, bur abrasion under water coolant, and conduction of all procedure by one experienced operator. All these factors can affect surface roughness, heat generation and phase transformation, and strength of zirconia specimens. According to Jum'ah et al. (2020), the contact pressure of bur cannot be standardized, but can affect the removal of material, and may be responsible for variations in the reported surface roughness of materials. In the present study, surface roughness was <0.2 µm in all groups. Bollen et al. (1996) showed significant enhancement of subgingival and supragingival plaque formation on zirconia crowns when their surface roughness exceeded 0.2 µm. In the present study, all surface treatments resulted in a surface roughness below the threshold of 0.2 µm, indicating their clinical acceptance. Jum'ah et al. (2020) reported higher surface roughness of sintered specimens with higher translucency (5Y‐TZP, 8Y‐TZP) compared with 3Y‐TZP, due to higher susceptibility of zirconia with higher translucency to bulk fracture, compared with 3Y‐TZP. However, in the present study, surface roughness did not significantly change by an increase in yttria content (higher translucency); although in general, type of zirconia and type of surface treatment had a significant interaction effect on surface roughness of zirconia.

In the present study, the surface roughness of bur abrasion + glazing and polishing + glazing subgroups (subgroups 4 and 5) was higher than that of the control group. In a study by Chun et al. (2017), the micrographs of glazed groups showed accumulation of glaze material in the form of islands, which resulted in higher surface roughness in some areas. As shown on SEM micrographs and profilometric assessments, glazing does not completely fix the surface defects caused by diamond bur abrasion.

In the current study, polishing of zirconia surface with EVE kit significantly decreased the surface roughness. Goo et al. (2016) reported a reduction in surface roughness after polishing with a polishing kit. In brief, rubber polishing alone appears to be the best protocol for conventional zirconia because it creates a smoother surface with higher resistance to fracture (Kolakarnprasert et al., 2019; Matsuzaki et al., 2015; Preis et al., 2011).

The translucency of the three zirconia types was significant different in all surface treatments. Translucency is determined by the amount of light absorbed, transferred, or reflected. In dental ceramics, translucency is affected by the chemical composition, crystalline content, and microstructure of ceramic (Spyropoulou et al., 2011). Translucency and optical properties of ceramics may significantly vary depending on ceramic type (Kim, 2020). Composilvan et al. (2018) reported that translucency improves by an increase in cubic phase content and depends on the structure of particles. Chun et al. (2017) indicated that differences between protocols and systems change the translucency, and microstructure and sintering can significantly affect the mechanical and optical properties of dental ceramics. Sen et al. (2018) reported that the microstructure of a material is an important parameter that can affect its physical and optical properties. The cubic phase of zirconia has isotropic crystalline orientation without light scattering at the grain boundaries, which increases the translucency (Zhang et al., 2016). Similarly, the present results showed higher translucency of Cube X2 zirconia, with cubic crystalline orientation. In the present study, no significant difference was found in the translucency of bur abrasion and polishing groups; however, reglazing in the majority of groups significantly increased the translucency compared with the control group, which was in agreement with the results of Manziuc et al. (2019). They reported that in zirconia specimens with lower thickness (0.8 mm), glazing decreased the translucency while in zirconia specimens with higher thickness (>0.8 mm), glazing increased the translucency. They also added that difference in the results can be due to optical interactions of the glaze layer with the ceramic mass. Since the specimen thickness was 1.2 mm in the present study, increased translucency after reglazing was in line with their results.

XRD in the current study revealed no significant change caused by the surface treatments. Chun et al. (2017) found that resin‐based diamond discs used for standardization of diamond bur abrasion did not cause phase transformation as analyzed by XRD. Their results were in line with the present findings.

Zirconia has a completely crystalline structure. Thus, its phase transformation can be optimally analyzed by XRD. Occlusal surface manipulation may result in heat generation and tetragonal to monoclinic phase transformation, as well as reverse transformation (Jum'ah et al., 2020; Mohammadi‐Bassir et al., 2017). Reglazing and heating of specimens at high temperature can be responsible for development of monoclinic phase after reglazing in subgroups 4 and 5 (bur abrasion + reglazing and polishing + reglazing) as shown by XRD.

With respect to phase transformation of zirconia after grinding and sandblasting, Karakoca and Yilmaz (Karakoca & Yılmaz, 2009) found that grinding had no significant effect on phase transformation. However, Lee et al. (2016) reported that grinding with diamond bur resulted in formation of small amounts of monoclinic phase in the zirconia. Variations in the reported results can be due to temperature rise during grinding.

Huh et al. (2016) and Al‐Haj Husain et al. (2016) reported that neither grinding nor polishing did not cause phase transformation in monolithic zirconia. Nonetheless, Park et al. (2017) showed that the volume of monoclinic phase increased by 0.09% following 8 min of polishing. However, in the present study, the polishing time was 1 min, which may be the reason for no crystalline phase transformation. Differences in methodology can explain variations in the results. A recent systematic review (Liu et al., 2023) reported that polishing rarely causes monoclinic phase transformation in zirconia while this phase transformation commonly occurs following grinding. If subsequent polishing is sufficient, the transformed monoclinic phase can be eliminated by removing the outermost superficial layer. They concluded that polishing is necessary to ensure optimal function and clinical service of zirconia (Liu et al., 2023).

This study had an in vitro design. Thus, generalization of results to the clinical setting must be done with caution.

## 5 CONCLUSION

Within the limitations of this in vitro study, the results showed that bur abrasion increased and polishing decreased the surface roughness of zirconia. Unlike expectations, reglazing did not create a smoother surface than polishing but increased the translucency of zirconia. Increasing the yttria content increased the translucency and relatively decreased the mechanical properties of zirconia. Of different surface treatments, polishing improved the surface properties and caused the smallest change in the mechanical properties of zirconia with different translucencies.

## AUTHOR CONTRIBUTIONS

*Study concept and design*: Fariba Ezoji. *Acquisition of data*: Maryam Jamali. *Analysis and interpretation of data*: Maryam Jamali. *Drafting of the manuscript*: Maryam Jamali. *Critical revision of the manuscript for important intellectual content*: Fariba Ezoji, Behnaz Esmaeili, and Maryam Jamali. *Statistical analysis*: Soraya Khafri. *Administrative, technical, and material support*: Maryam Jamali. *Study supervision*: Fariba Ezoji, Behnaz Esmaeili, Soraya Khafri, and Maryam Jamali.

## CONFLICT OF INTEREST STATEMENT

The authors declare no conflict of interest.

## Data Availability

The data supporting the findings of our recent study are available in the supplementary material of our article.
